# Challenges and Opportunities of Precision Medicine in Sickle Cell Disease: Novel European Approach by GenoMed4All Consortium and ERN-EuroBloodNet

**DOI:** 10.1097/HS9.0000000000000844

**Published:** 2023-02-22

**Authors:** Anna Collado, Maria Paola Boaro, Sigrid van der Veen, Amira Idrizovic, Bart J. Biemond, David Beneitez Pastor, Ana Ortuño, Elena Cela, Anna Ruiz-Llobet, Pablo Bartolucci, Marianne de Montalembert, Gastone Castellani, Riccardo Biondi, Renzo Manara, Tiziana Sanavia, Piero Fariselli, Petros Kountouris, Marina Kleanthous, Federico Alvarez, Santiago Zazo, Raffaella Colombatti, Eduard J. van Beers, María del Mar Mañú-Pereira

**Affiliations:** 1 Service of Pediatric Hematology, Hospital Universitari Vall d’Hebrón, ERN-EuroBloodNet member, Barcelona, Spain; 2 Rare Anemia Disorders Research Laboratory, Cancer and Blood Disorders in Children, Vall d’Hebrón Institut de Recerca, Barcelona, Spain; 3 Department of Women’s and Children’s Health, University of Padova, ERN-EuroBloodNet member, Padova, Italy; 4 Center for Benign Haematology, Thrombosis and Haemostasis, Van Creveldkliniek, University Medical Center Utrecht, Utrecht University, ERN-EuroBloodNet member, Utrecht, the Netherlands; 5 Department of Hematology, Amsterdam UMC, University of Amsterdam, ERN-EuroBloodNet member, Amsterdam, the Netherlands; 6 Department of Hematology, Hospital Universitari Vall d’Hebrón, ERN-EuroBloodNet member, Barcelona, Spain; 7 Pediatric Hematology and Oncology Unit, Hospital Gregorio Marañón, Universidad Complutense de Madrid, ERN-EuroBloodNet member, Madrid, Spain; 8 Service of Pediatric Hematology, Hospital Sant Joan de Déu, Universitat de Barcelona, Institut de Recerca Hospital Sant Joan de Déu, ERN-EuroBloodNet member, Barcelona, Spain; 9 Sickle Cell Coordinating Referral Center, Henri Mondor, IMRB, ERN-EuroBloodNet member, Paris, France; 10 Sickle Cell Center, Hôpital Universitaire Necker-Enfants maladies, INSERM 1134 INTS, ERN-EuroBloodNet member, Paris, France; 11 Dipartimento di Medicina Specialistica, Diagnostica e Sperimentale, Univeristà di Bologna, Italy; 12 Department of Neuroscience, University of Padova, ERN-EuroBloodNet member, Padova, Italy; 13 Department of Medical Sciences, University of Torino, Italy; 14 Molecular Genetics Thalassaemia Department, The Cyprus Institute of Neurology and Genetics, Nicosia, Cyprus; 15 Information Processing and Telecommunications Center, Universidad Politécnica de Madrid, Spain

We read with great interest the publication by El Hoss et al^1^ in *HemaSphere* about precision medicine and sickle cell disease (SCD). The authors give an excellent overview of the enormous clinical variability in SCD and outline the main challenges for precision medicine. Our comment adds some considerations that hamper the large-scale generation of good-quality data to advance research in SCD. We also introduce the novel European approach by GenoMed4All Consortium to overcome some of these challenges.

The authors acknowledge that the lack of high-quality evidence and good characterization of patient groups makes it difficult to achieve personalized clinical practice. The use of transcranial Doppler ultrasound assessment to identify children at increased risk of stroke is perhaps the best example of precision medicine in SCD. Nevertheless, a survey conducted by the ERN-EuroBloodNet showed that more than 70% of SCD children in European expert centers are either not or incorrectly screened according to guidelines.^2^ This results in incomplete clinical data and incorrect risk classification, which is a source of bias in any observational study regarding stroke in SCD. In addition, ancestry plays an important role in disease risk, and in responses to environmental exposures and drug therapies. Research conducted in SCD considers standards and references developed in mostly white populations decreasing accuracy of genome-wide association studies (GWAS) studies when enriched by imputation approaches and the interpretability of metabolites analysis normalized to healthy white population controls.^3,4^

As pointed out by the authors, the variability in SCD expression is only partially explained by genetics. Metabolomics, resulting from the combination of genomic, transcriptomic, proteomic and oxidative changes, respond faster to external stimuli than any other “omics.” Metabolomics is thereby especially useful for the surveillance of the metabolic profile of the red blood cells (RBCs), in which gene-expression profiling is not an option. Emerging evidence suggests that RBC glycolytic intermediates, for example, 2,3-DPG and ATP are among the strongest factors that influence sickling. Many other metabolic pathways that orchestrate disease severity in SCD, such as arginine, ornithine, and citrulline plasma levels have been identified previously by metabolomics profiling. Therefore, untargeted metabolomics could be a strong biomarker of SCD severity. Furthermore, for many of the identified metabolic targets, new therapies are under development and many more metabolic druggable targets are in early-stage development.^5^

SCD research in Europe is limited by the relatively small, variable, and widely dispersed patient cohorts. In addition, the lack of harmonization among the repositories hampers world-wide collaborations. Artificial Intelligence (AI) can aid in analyzing large quantities of data, but only if it is collected and organized in a standardized way. To overcome some of the presented issues, GenoMed4All^6^ is a European initiative to provide personalized solutions for hematological diseases’ control and prevention by exploiting the power of AI. This is achieved by pooling “-omics,” clinical and imaging data from different expert centers through a secure federated learning platform, which encapsulates different prediction models implemented by machine learning algorithms. The advantage of a federated learning approach is to share among the centers only the “learned parameters” from AI algorithms (eg, weights of Neural Networks) of the models, preserving the data privacy and keeping the data in the original repositories. This is essential for any rare disease (RD) platform as the strong privacy legislation makes sharing health-related data across the European Union (EU) borders increasingly cumbersome. On top of this, innovative AI models, based also on new deep learning approaches scaled up by high-performance computing, will allow the definition of new predictive risk scores to enhance the clinical prevention by boosting the processing capacity of data repositories in the EU, thus empowering data analysis and forward-thinking research. So far, GenoMed4All has gathered 10 starting partners from ERN-EuroBloodNet and it includes 3 use cases: multiple myeloma, myelodysplastic syndrome, and SCD.

The Rare Anaemia Disorders European Epidemiological Platform (RADeep),^7^ an initiative endorsed by ERN-EuroBloodNet’s ENROL registry, supports GenoMed4All in data standardization for both centralized and federated data processing through AI models. In line with this, GenoMed4All has made an exceptional effort to harmonize data collection to provide accurate and unambiguous definitions, including international codifications, of more than 400 core variables also mapped in the common data model Observational Medical Outcomes Partnership (OMOP) to ensure repositories’ interoperability. Other initiatives, as Harmony,^8^ a European Public-Private Partnership for Big Data in Hematology, also implements OMOP, enabling interoperability in the context of Genomed4All for myelodysplastic syndromes. Table 1 lists some of the challenges raised by El Hoss et al^1^ and how GenoMed4All plans to address them.

**Table 1:** Challenges of Precision Medicine in SCD and GenoMed4All Approach to Address Them

Issues Raised on Personalized Medicine for SCD	How Genomed4ALL Addresses Them
**Can we identify prognostic groups in SCD**	
Different types of SCD/co-inheritance of alpha thalassemia.	Inclusion criteria: SCD patients with all genotypes. Genetic analysis of *HBB* and *HBA2 and HBA1* genes is standardized for full coverage of mutations.
Variation in Hb F levels. Mutations on *HBF2*, *BCL11A*, and *HBS1L-MYB* genes. Influence of other genetic factors (known or unknown).	A GWAS analysis will be performed on all patients in a standardized way and enriched by imputation.
Blood tests and biomarkers.	Parameters agreed in consensus will be collected at steady state for all patients in a standardized form.
Imaging	TCD and MRI results will be collected for the most recent evaluations.
**Selection of different treatments in sickle cell disease**	
Drugs promoting HbF synthesis. Hydroxyurea is started at an early age and as a standard of care for most patients, making it difficult to assess the effect on the evolution of the disease among different groups of patients all receiving this drug.	Use of hydroxyurea at the time of inclusion and total cumulative years of use. MCV and % of hemoglobin F in steady state are also registered. The GWAS analysis is expected to cover the presence of particular variants in the *BCL11A*, *HMIP* and *HBC2* genes. The inclusion of metabolomics data will shed light on the effect of hydroxyurea on different patients.
New drugs with different targets (eg, Increasing hemoglobin levels, blocking abnormal red cell adhesion).	All participation in clinical trials regarding new drugs will be collected.
Drugs targeting abnormal erythropoiesis and bone marrow niche. Bone marrow aspirates are not performed routinely in SCD patients.	IE by the assessment of soluble transferrin receptor, absolute reticulocyte count, and transfusion status (either occasional or regular) will be evaluated in all the patients.

GWAS = genome-wide association study; HbF = hemoglobin F; IE = ineffective erythropoiesis; MCV = mean corpuscular volume; MRI = magnetic resonance imaging; SCD = sickle cell disease; TCD = transcranial Doppler.

In addition to “-omics” and clinical data, for the SCD use case GenoMed4All is collecting also anonymized magnetic resonance imaging (MRI) data already available in European Centers of the ERN-EuroBloodNet Network to improve the identification of silent cerebral infarcts (SCI) through AI models.^9^ A stepwise procedure allowed the optimization of SCI identification, with distinction between SCI and non-clinically significant background noise or other white matter hyperintensities. Radiomics and AI will offer the opportunity to harness the potential of big data analysis to understand the natural history of SCD and optimize diagnostics for chronic complications such as SCI, while reducing variability across centers and in healthcare access.

Socioeconomic, psychological, and, to some extent, environmental factors can also be systematically gathered by the implementation of validated “patient reported outcomes measures” (PROMs). ERICA Consortium^10^ has recently released a PROMs repository as a first attempt to identify and centralize PROMs in RDs. PROMs are of special interest in SCD, especially following the COVID-19 pandemic, to assess disease burden (eg, pain not requiring emergency consultation) that is not captured in hospital clinical records. In recent years, the interest in using them as an additional monitoring tool in routine clinical practice is increasing, as it gives structured and reliable information on the patient’s global health status from a standpoint never used before. Moreover, these initiatives are also strongly endorsed by patient advocacy groups. The RADeep consortium will incorporate PROMs in a second phase of implementation; it will enable in the future to include PROMs as a complementary source data for AI-based predictive risk scores, thus considering other socioeconomics and psychological factors into the analysis.

The GenoMed4All SCD use case aims to develop AI-based predictive risk scores, overall and over time, for the most prevalent and severe clinical outcomes (eg, stroke, vaso-occlusive crisis, acute chest syndrome, organ damage). This will result in the first European large multi-modal dataset including clinical, laboratory, imaging (cerebral MRI), oxygenscan, genetics data, and metabolomics for at least 1.000 SCD patients. The challenges for precision medicine in SCD are vast but the GenoMed4all consortium paves the way to advance research in the EU.

## AUTHOR CONTRIBUTIONS

AC and MdMM-P prepared the first draft document. RC, EvB, and MdMM-P prepared the final document. All authors contributed to the draft document and review the final document. EvB and MdMM-P equally contributed to the final document as last authors.

## DISCLOSURES

The authors have no conflicts of interest to disclose.

## SOURCES OF FUNDING

GA 101017549 – GENOMED4ALL - H2020-SC1-FA-DTS-2020-1. ‘Genomics and Personalized Medicine for all though Artificial Intelligence in Haematological Diseases’. Instituto de Salud Carlos III through the project ‘PI20/01454: ENABLING PERSONALIZED MEDICINE OF SICKLE CELL DISEASE PATIENTS BASED ON INTEGRATIVE DIAGNOSIS OF NEW GENERATION METHODOLOGIES’ (Co-funded by European Regional Development Fund/European Social Fund; ‘A way to make Europe’/’Investing in your future”). GA 101085717 - ERN-EuroBloodNet – EU4H-2022-ERN-IBA-01 - European Reference Network on Rare hematological diseases – ERN-EuroBloodNet.

## References

[1] El Hoss S El Nemer W Rees DC. Precision medicine and sickle cell disease. HemaSphere. 2022;6:e762. 35999951 10.1097/HS9.0000000000000762 PMC9390823

[2] Cuzzubbo D Gutierrez-Valle V Casale M. Limited access to transcranial Doppler screening and stroke prevention for children with sickle cell disease in Europe: results of a multinational eurobloodnet survey. Blood. 2021;138(Suppl 1):915. 38984411 10.1002/pbc.31190

[3] Vergara C Parker MM Franco L. Genotype imputation performance of three reference panels using African ancestry individuals. Hum Genet. 2018;137:281–292. 29637265 10.1007/s00439-018-1881-4 PMC6209094

[4] Beutler E. Red Cell Metabolism: A Manual of Biochemical Methods. 3rd ed. Orlando, FL, USA: Grune & Stratton; 1984.

[5] Adebiyi MG Manalo JM Xia Y. Metabolomic and molecular insights into sickle cell disease and innovative therapies. Blood Adv. 2019;3:1347–1355. 31015210 10.1182/bloodadvances.2018030619 PMC6482357

[6] Genomed4All Consortium. https://genomed4all.eu/. Accessed December 30, 2022.

[7] RADeep Consortium. https://www.radeepnetwork.eu/. Accessed December 30, 2022.

[8] HARMONY Consortium. https://www.harmony-alliance.eu/. Accessed December 30, 2022.

[9] Boaro MP Biondi R Biondini N. S265: Radiomics and artificial intelligence for identification and monitoring of silent cerebral infarcts in sickle cell disease: first analysis from the GenoMed4All European Project. HemaSphere. 2022;6(S3):166–167.

[10] ERICA Consortium. https://erica-rd.eu/. Accessed December 30, 2022.

